# Immunoproteasome deficiency alters microglial cytokine response and improves cognitive deficits in Alzheimer’s disease-like APPPS1 mice

**DOI:** 10.1186/s40478-017-0453-5

**Published:** 2017-06-24

**Authors:** Lisa K. Wagner, Kate E. Gilling, Eileen Schormann, Peter M. Kloetzel, Frank L. Heppner, Elke Krüger, Stefan Prokop

**Affiliations:** 1 Department of Neuropathology, Charité – Universitätsmedizin Berlin, Charitéplatz 1, 10117 Berlin, Germany; 2 Present Address: Department of Neurology, Brigham and Women’s Hospital, Harvard Medical School, Boston, MA USA; 3 Institute of Biochemistry, Charité – Universitätsmedizin Berlin, Charitéplatz 1, 10117 Berlin, Germany; 4 Cluster of Excellence, NeuroCure, Charitéplatz 1, 10117 Berlin, Germany; 5 Berlin Institute of Health (BIH), Berlin, Germany; 6 Present Address: Institute of Medical Biochemistry and Molecular Biology, University Medicine Greifswald, Ferdinand-Sauerbruch-Str./ DZ7, 17475 Greifswald, Germany; 7 Present Address: Department of Pathology and Laboratory Medicine, Hospital of the University of Pennsylvania, Philadelphia, PA USA

**Keywords:** Proteasome, Immunoproteasome, Microglia, Inflammation, Alzheimer’s disease

## Abstract

The immunoproteasome (iP) represents a specialized type of proteasomes, which plays an important role in the clearance of oxidant-damaged proteins under inflammatory and pathological conditions determining the outcome of various diseases. In Alzheimer’s disease (AD)-like APPPS1 mice Aβ-deposition is paralleled by iP upregulation, most likely mediated through type I interferon induction. To define the impact of increased iP expression we crossed APPPS1 mice with mice deficient in the iP subunit LMP7 resulting in impaired iP function. While LMP7 deficient APPPS1 mice showed no major change in cerebral Aβ-pathology, we observed an altered cytokine response in microglia isolated from LMP7 deficient APPPS1 mice compared to LMP7 expressing APPPS1 control mice. The altered microglial cytokine profile upon iP deficiency in the presence of extracellular Aβ-pathology was associated with an improvement of Aβ-associated cognitive deficits typically present in APPPS1 mice. Our findings suggest a role for iP in the regulation of the innate immune response towards extracellular Aβ-pathology and indicate that inhibition of iP function can modulate the cognitive phenotype upon overexpression of Aβ.

## Significance statement

We herein show that the immunoproteasome (iP) significantly modulates microglial pro-inflammatory cytokine secretion in a murine model of AD and alters the pathology-associated behavioral phenotype in these mice. Since iP impairment did not affect Aβ plaque burden our data demonstrate that inflammation has a critical effect on cognitive outcome independent of Aβ-pathology. Our study links iP function, the innate immune response and cognitive performance for the first time in an in vivo model of neurodegeneration and suggests that modulation of the iP is a viable option for the treatment of AD.

## Introduction

The ubiquitin-proteasome system (UPS) is a major regulator of protein homeostasis, vital to regulatory processes via degradation of short-lived proteins involved in the cell cycle, differentiation, transcriptional regulation or apoptosis, and is also important for the degradation of misfolded and damaged proteins [22, 28]. Proteins destined for degradation are labelled with a poly-ubiquitin tag, which is recognized by the 19S regulatory cap of the 26S proteasome and broken down by the 20S core containing the three catalytically active β1, β2 and β5 subunits. The immunoproteasome (iP) is an isoform, which is constitutively expressed in immune cells and induced by pro-inflammatory cytokines such as type I and type II interferons (IFNs) in almost any other cell type. IFN signaling leads to incorporation of the alternative catalytically activite β subunits β1i/LMP2, β2i/MECL-1 and β5i/LMP7 into newly formed iPs [1, 28]. There are subtypes of iPs which contain only one or two βi subunits [54], however the β5i/LMP7 subunit is indispensable for iP formation [40]. Depending on the tissue and specific cells types, different proteasome compositions are expressed and can coexist [7]. Previous studies have demonstrated that iPs possess enhanced overall activity compared to standard proteasomes [49] and extended the role of iPs; under conditions of cellular stress and inflammation, the inducible forms of proteasomes were shown to be vital for the degradation of misfolded and oxidant-damaged proteins to prevent disease progression [40, 49]. Moreover, patients harboring mutations in proteasome subunit genes that cause proteasome associated autoinflammatory syndromes (PRAAS) with proteasome dysfunction combined with concomitant proteotoxic stress, exhibit increased type I IFN production [7, 8].

Notably, the UPS also appears to be implicated in the pathogenesis of neurodegenerative diseases [3, 12, 15] such as Alzheimer’s disease (AD), the most common neurodegenerative disorder [44]. Previous work has shown that ubiquitinylated protein deposits accumulate in the brains and cerebrospinal fluid of AD patients [19, 24, 34, 38, 42, 56, 58] and in rodent models of disease [33]. A malfunction of the UPS was reported in AD patients [27] and mouse models involving extracellular beta-amyloid (Aβ) deposits [41, 48]. However, it is still unclear whether the presence of Aβ leads to proteasomal impairment or if disrupted proteasome activity enhances cellular toxicity. Besides intense investigations on the function of the standard proteasome, recent publications demonstrate an upregulation of the iP in microglia and astrocytes surrounding Aβ plaques in a mouse model of AD, as well as positive correlation of iP activity with increasing severity of tau pathology in AD patients [2, 41]. However, the precise role of iPs in regulating the innate immune response towards Aβ deposits and a potential impact of a modulation of iP activation on disease course and cognitive function has not been explored in vivo so far.

To pinpoint the involvement of the iP in Aβ-pathology, we analyzed the expression of iP subunits during the course of normal aging and in AD-like pathology in APPPS1 mice [45]. To further dissect the role of iPs in AD-like pathology, we crossed APPPS1 mice to β5i/LMP7 deficient mice lacking exons 1–5 of the proteasome subunit beta type 8 (*PSMB8*) gene, which encodes for the catalytic iP subunit β5i/LMP7 and is inevitable for iP formation, resulting in a loss of iP assembly in LMP7 deficient mice [18].

Here we show that iP expression is increased upon aging and accelerated by the onset of Aβ-pathology. While the lack of LMP7 had no impact on the development and progression of Aβ burden in APPPS1 mice, the pattern of cytokines secreted by microglia was significantly altered, resulting in an ameloriation of cognitive deficits typically found in APPPS1 mice. These data suggest that iPs contribute to the regulation of Aβ-driven innate immune responses and modulate cognitive deficits associated with AD pathology.

## Materials and methods

### Animals and tissue collection

APPPS1 mice harboring the Swedish amyloid precursor protein (APP) mutation KM670/671NL in conjunction with the presenilin 1 mutation L166P [45] were crossed to β5i/LMP7 deficient mice [18], lacking exons 1 to 5 of proteasome (prosome, macropain) subunit beta type 8 *(PSMB8)* gene, that encodes for LMP7. All experiments used littermate mice of both genders. Mice were group housed under pathogen–free conditions on a 12 h light/dark cycle, and food and water were provided to the mice ad libitum. All animal experiments were performed in accordance to the national animal protection guidelines approved by the regional offices for health and social services in Berlin (LaGeSo). Animals were euthanized and transcardially perfused with 1× phosphate buffered saline (PBS). Brains were carefully removed and fixed in 4% paraformaldehyde (PFA) for 2 days followed by immersion in 30% sucrose for at least 1 day for immunohistochemical analysis or was snap-frozen in a 2-methylbutane (Merck) bath placed in liquid nitrogen for subsequent processing.

### Real time RT- PCR

For isolation of RNA, brain tissue was homogenized in TRIzol® (Life Technologies) and centrifuged for 10 min at 12,000 x g (4 °C). The supernatant was mixed with chloroform, vigorously mixed and incubated for 3 min at room temperature. Samples were centrifuged for 15 min at 12,000 x g (4 °C) and the supernatant carefully removed, mixed with chilled isopropanol and incubated for 10 min at room temperature before a further 30 min centrifugation step at 12,000 x g (4 °C). The resulting pellet was dissolved in ethanol, centrifuged for 10 min at 7500 x g (4 °C), and ethanol removed. This step repeated and the dry pellet dissolved in ultrapure H_2_O. cDNA synthesis was performed using the Transcriptor High Fidelity cDNA Synthesis kit (Roche) according to the manufacturer’s instructions.

Real time PCR using TaqMan gene expression assays (Applied Biosystems) for *Hprt*, *PSMB9* (encoding LMP2); *PSMB8* (encoding LMP7), *Ifn-α*, *Ifn-β*, *Isg15* and *Cxcl10* (IP-10) were performed using a Rotor-Gene RG-3000 (Corbett Research).

### Proteasome activity assay

Brain tissue was homogenized in TSDG buffer (10 mM Tris pH 7.0, 10 mM NaCl, 25 mM KCl, 1.1 mM MgCl2, 0.1 mM EDTA, 1 mM DTT, 2 mM ATP, 10% glycerin) and underwent 5 cycles of freezing and defrosting using liquid nitrogen. Samples were centrifuged for 60 min at 13,000 x g (4 °C) und protein concentration determined using the Pierce BCA Protein Assay Kit (Thermo Fischer) according to the manufacturers protocol. Samples were loaded onto a 96-well plate followed by the addition of the substrate Suc-Leu-Leu-Val-Tyr-AMC (Bachem). Plates were incubated at 37 °C for 90 min and fluorescence recorded using a Synergy-HT (Bio Tek) plate reader. To exclude non-proteasomal substrate degradation, samples were incubated for 10 min with epoxomicin (1 μM; 37 °C) before loading on the plate and values were substracted from lysates incubated with DMSO control. A standard sample of purified 26S standard proteasome was loaded onto each plate, and measurements from samples were thereafter corrected against this control.

### Immunohistochemical staining

Frozen tissue was cut in 30 μm thick sections and stored free floating in cryoprotectant solution (30% ethylenglycol, 20% glycerol, 50 mM sodium phosphate buffer, pH 7.4) at 4 °C until further use. For immunohistological staining, sections were rinsed in 1× PBS, incubated in blocking buffer (1× PBS containing 0,3% Triton X-100 and 10% normal goat serum) for 1 h at RT and primary antibodies for 4G8 (1:1000; Covance) and Iba-1 (1:500; Wako Chemicals) were diluted in 1× PBS/ 0.3% triton X-100/ 5% normal goat serum and incubated over night at 4 °C. Sections were washed with 1× PBS to wash off excessive primary antibodies, incubated with species specific peroxidase-coupled secondary antibodies (goat anti-mouse or goat anti-rabbit (1:300, Dianova)) diluted in 1× PBS/ 0.3% Triton X-100/ 5% normal goat serum and incubated for 1 h on a shaker at RT before developed with liquid diaminobezadine (DAB) (Dako, K3647). Sections were counterstained with matured hematoxylin followed by dehydration in an ascending alcohol series before covered using Roti®-Histokitt II mounting medium.

For Congo red staining, cerebral free floating sections were mounted on glass slides. Sections were incubated in stock solution I (0.5 M NaCl in 80% ethanol, 1% NaOH) for 20 min and in stock solution II (8.6 mM Congo red in stock solution I, 1% NaOH) for 45 min. After rinsing twice in absolute ethanol, sections were counterstained with mature hematoxylin and dehydrated in ascending alcohol series, twice rinsed in 98% xylene for 1 min, before mounting using Roti®-Histokitt II mounting medium. Light microscopy and stereology were performed using a Stereo Investigator system (MicroBrightField) and DV-47d camera (MicroBrightField) mounted on an Olympus BX53 microscope (Olympus, Germany). Fluorescence imaging was performed using an Olympus XM10 monochrome fluorescence CCD camera (Olympus, Germany).

### Stereological quantification

Quantitative analyses of Aβ plaque load and numbers in cerebral cortical sections were performed using the Stereo Investigator system including an Olympus microscope BX53, the QImaging camera COLOR 12 BIT and a stage controller MAC 6000. For analyses, the Stereo Investigator 64-bit software (MBF Bioscience) was used. Cortical Aβ plaque burden as assessed by 4G8 or Congo red staining was quantified with the Area Fraction Fractionator method of the Stereo Investigator software as previously described [55]. Briefly, the area covered by Aβ was quantified using the following settings: counting frame size 90 × 90 μm, scan grid size 400 × 500 μm and Cavallieri grid spacing 10 μm. Iba-1+ microglia area covered was assessed with cellSens software (Olympus) and the cortical region of interest was automatically analyzed according to manufacturer’s instructions.

### Protein extraction

Frozen brain tissue was homogenized according to a 4-step extraction method as described in [25] with slight modifications. In brief, hemispheres were homogenized consecutively in Tris buffered saline (TBS buffer) (20 mM Tris, 137 mM NaCl, pH = 7.6), followed by a 45 min centrifugation step at 100,000 x g (4 °C). The supernatant was collected as the Tris soluble fraction and the pellet was resuspended in Triton-X buffer (TBS buffer containing 1% Triton X-100). This was followed by further identical centrifugation and resuspension procedure and this cycle was repeated with SDS buffer (2% SDS in ddH_2_O) and formic acid (FA; 70% formic acid in ddH_2_O). Immediately before use, protease inhibitors (Roche, 1 tablet per 10 ml) and a phosphatase inhibitor cocktail 3 (Sigma) were added to the first two buffers. Brain extracts were incubated 30 min on ice (except SDS and FA homogenates, which was incubated at RT) after resupending before centrifugation. Protein concentrations of each fraction were determined using the Quantipro BCA Protein Assay Kit (Pierce) according to the manufacturers protocol using the Tecan Infinite® 200 M photometer (Tecan).

### Immunoblot and native PAGE analysis

Expression levels of endogenous mouse and transgenic human APP and major C-terminal cleavage products of APP (CTFα and CTF) and LMP7 iP subunits were assessed by Western blot analysis according standard protocols [55]. SDS fractions of brain homogenates described above were analyzed using primary antibodies against β5i/LMP7 (pc, K63, labstock generated against peptides of LMP7 protein; 1:5000; Prof. Peter M. Kloetzel, Institute of Biochemistry, Charité – Universitätsmedizin Berlin, Charitéplatz 1, 10,117 Berlin, Germany), APPct (Sigma, A8717); 1:1000) and GAPDH (Santa Cruz; 1:2000). An HRP-conjugated anti-rabbit IgG antibody (GE healthcare) was used as secondary antibody and immunoreactive bands were visualized using the Amersham ECL immunoblotting detection system (GE healthcare). For native PAGE analysis, tissue was homogenized in TSDG buffer (10 mM Tris pH 7.0, 10 mM NaCl, 25 mM KCl, 1.1 mM MgCl2, 0.1 mM EDTA, 1 mM DTT, 2 mM ATP, 10% glycerin) and extracts loaded onto precast native PAGE gels (3%–12%, Invitrogen).

### Detection of Aβ_1–40_ and Aβ_1–42_ species and cytokines by multiplex mesoscale assays MSD

Aβ_1__–__40_ and Aβ_1–42_ and cytokine concentrations in brain extracts and cell culture supernatants were determined with the MSD 96-Well MULTI-SPOT® Human (6E10) Abeta Triplex Assay (Meso Scale Discovery) or the V-PLEX™ Mouse Cytokine Assay according to manufacturer’s instructions and analyzed on a SECTOR Imager 6000 plate reader (Meso Scale Discovery).

### Microglia cell isolation and stimulation

For cell isolation procedure, the whole brain was placed in HBSS on ice and further processed with neuronal kit dissociation according to manufactures instructions.

Isolation of CD11b^+^ cells from brain tissue was performed using the Neural Tissue Dissociation Kit (P) (Miltenyi Biotech) and the magnetic cell sorting (MACS) technique using CD11b-labeled magnetic Microbeads (Miltenyi Biotech) according to manufacturer’s instructions.

50.000 CD11b^+^ microglia were cultured overnight in a 96 well plate. Culture medium was removed and LPS (1 μg/ml) diluted in serum-free culture medium was added to stimulate the cells. Equivalent amount of PBS in serum-free medium was used as stimulation negative control. Supernatant for baseline measurements were collected prior to LPS stimulation. After 14 h, medium was collected, snap frozen in liquid nitrogen and stored at −80 °C for further cytokine analysis as described above.

### Behavioral analysis

Cognition was assessed using the novel object recognition task (NOR) and the Barnes maze test at the age of 250 days. Experimenters were blinded during testing and data acquisition. All tests were performed in the animals’ active phase in sound proof testing chambers with controlled temperature and humidity at the Berlin Animal Outcome Unit (NeuroCure Cluster of Excellence, Berlin, Germany). Experiments were conducted in a sound-attenuated testing chamber with the illumination set at 30–40 lx. Mice were allowed to acclimate to the testing area for at least 30 min prior to testing. Each animal was allowed to explore freely for 5 min and activity was recorded with an overhead camera using an automated system (Viewer III Version 3.0.1.205, Biobserve, St. Augustin, Germany). Animals were returned to their home cage at the end of the trial. Twenty-four hours after habituation, the animals were exposed to the familiar arena with two identical objects placed at an equal distance and allowed to explore for 5 min. On the 3rd day, one of the familiar objects was exchanged for a different, novel object and the mice were allowed to explore the Open field in the presence of the familiar and novel objects for 5 min. The time spent exploring each object and the number of visits to each object was recorded using an automated system (Viewer III Version 3.0.1.205, Biobserve, St. Augustin, Germany).

An elevated Barnes maze apparatus (TSE Systems GmbH, Bad Homburg, Germany; diameter 920 mm) containing 19 empty holes and one hole with a hidden escape chamber was used for testing spatial learning and memory. Animals were trained for the Barnes maze task for 4 days prior to testing. Each animal received 4 trials per day, spaced at 15 min intervals for each of the 4 days in order to learn the task. Extra-maze visual cues were placed around the room and remained consistent throughout the training and testing phase. During training, animals were allowed to freely explore for 3 min per trial. Bright lights (75–85 lx) and a loud white noise were used to motivate the animals to locate the escape box. The number of errors (nose-pokes into incorrect holes) and the latency to reach the target (hole with escape box) was scored. To test short-term spatial memory retention, one 90-s trial was conducted on day 5 wherein the escape box was removed. The time to reach the target hole (latency to target) and time spent in the target zone was measured. To test long-term memory retention, another 90-s trial was conducted 7 days later. Behavior was recorded using an overhead camera and automated software system (Viewer III Version 3.0.1.205, Biobserve, St. Augustin, Germany).

### Statistics

Statistical analyses were performed using the GraphPad Prism 6 Software. Differences between two groups were evaluated by Student’s t-test or Mann-Whitney test for pairwise comparison of experimental groups or by one-way ANOVA or two-way ANOVA with Bonferroni post-tests for comparison of more than two experimental groups, as indicated. Data are represented as means +/− SEM. Statistical significance is indicated as follows: * *p* < 0.05, ** *p* < 0.01 and *** *p* < 0.001.

## Results

### Expression of iP subunits is increased during aging and is accelerated by Aβ-pathology in APPPS1 mice

**Fig. 1:**
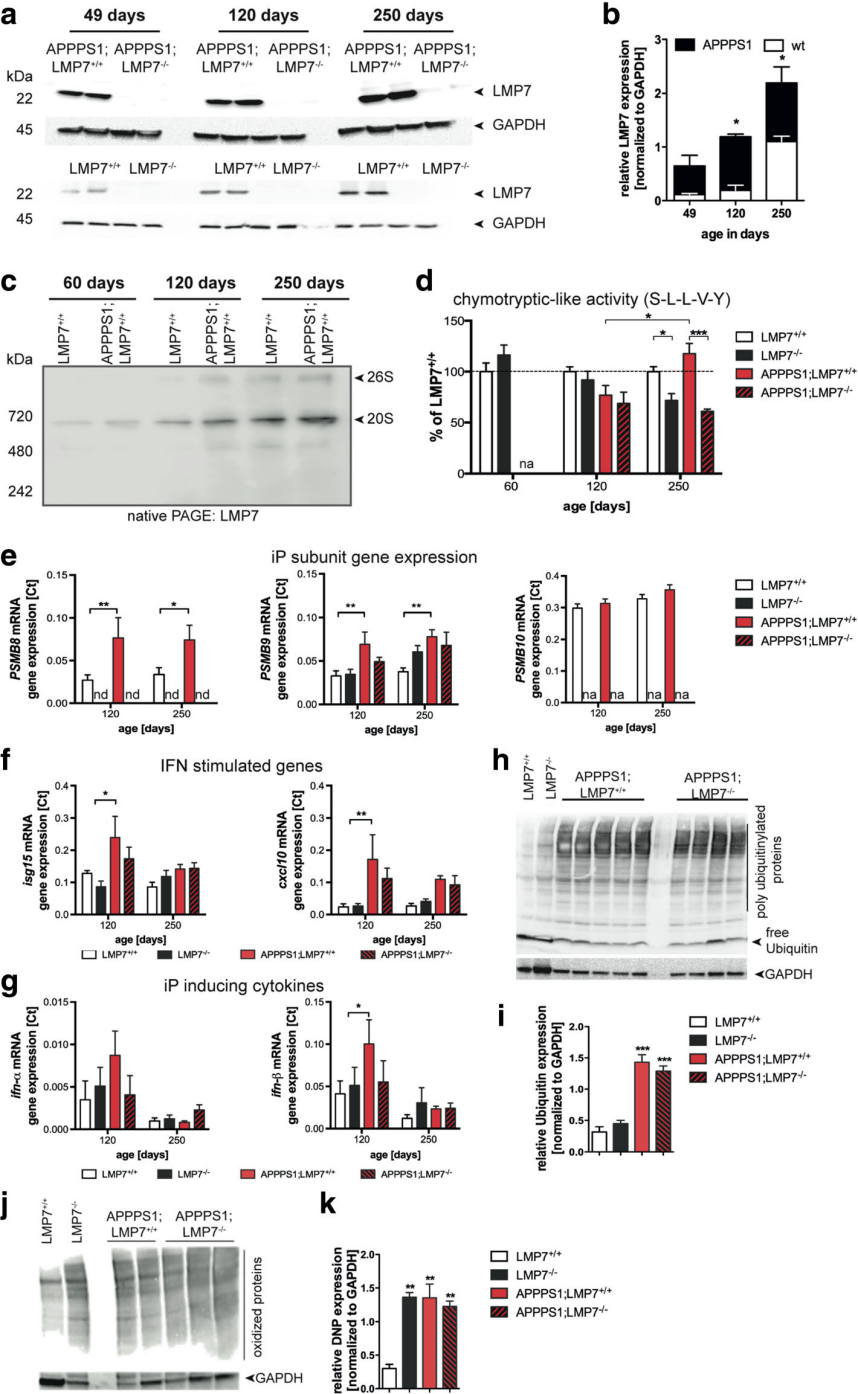
iP subunit expression in the brain is increased upon aging and enhanced in APPPS1 mice. **a﻿ **Western blot analysis of LMP7 expression in brain homogenates of 49d, 120d and 250d old wildtype (LMP7^+/+^) and APPPS1;LMP7^+/+^ mice (respective littermate control LMP7^−/−^ and APPPS1;LMP7^−/−^ mice served as a control for antibody specificity) and (**b**) corresponding densitometric quantification (right panel; *n* = 3–5 mice per group, (LMP7^+/+^: 49d vs. 250d; *p* = 0.0253; LMP7^+/+^ vs. APPPS1;LMP7^+/+^: 49d ns; 120d *p* = 0.0222; 250d *p* = 0.0065; two-way ANOVA followed by Bonferroni post-tests). **c** Native PAGE analysis of brain homogenates from LMP7^+/+^ and APPPS1;LMP7^+/+^ mice. Proteasome complexes were visualized by western blotting for the iP subunit LMP7. **d** Total 26S chymotryptic peptide-hydrolyzing activity was analyzed in brain homogenates from LMP7^+/+^, LMP7^−/−^, APPPS1;LMP7^+/+^ and respective littermate APPPS1;LMP7^−/−^ mice using Suc-LLVY-AMC peptide hydrolysis (*n* = 5 per group; LMP7^+/+^ vs. LMP7^−/−^: *p* = 0.0061; APPPS1;LMP7^+/+^ vs. APPPS1;LMP7^−/−^: *p* = 0.0001; two-way ANOVA followed by Bonferroni post-tests). **e**-**g** Quantitative PCR analysis of *PSMB8*, *PSMB9*, *PSMB10*, *isg15*, *cxcl10, Ifn-α* and *Ifn-β* from whole brain tissue of LMP7^+/+^, LMP7^−/−^, APPPS1;LMP7^+/+^ and respective littermate APPPS1;LMP7^−/−^ mice (*n* = 5 per group; *PSMB8* 120d: LMP7^+/+^ vs. APPPS1;LMP7^+/+^: *p* = 0.0020; 250d: LMP7^+/+^ vs. APPPS1;LMP7^+/+^: *p* = 0.0162; *PSMB9* 120d: LMP7^+/+^ vs. APPPS1;LMP7^+/+^: *p* = 0.0041; 250d: LMP7^+/+^ vs. APPPS1;LMP7^+/+^: *p* = 0.0014; *isg15* 120d: LMP7^+/+^ vs. APPPS1;LMP7^+/+^: *p* = 0.0165; *cxcl10* 120d: LMP7^+/+^ vs. APPPS1;LMP7^+/+^: *p* = 0.0007; *ifn-β*: LMP7^+/+^ vs. APPPS1;LMP7^+/+^: *p* = 0.0410; two-way ANOVA followed by Bonferroni post-tests). **h** Western blot analysis of poly-ubiquitin conjugates in brain homogenates of 120 days old APPPS1;LMP7^+/+^ and respective littermate APPPS1;LMP7^−/−^ mice as well as age-matched wild-type and LMP7^−/−^ mice and (**b**) corresponding densitometric quantification (*n* = 3–5 mice per group; *** *p* = 0.001, one-way ANOVA followed by Bonferroni post-tests). (**j**) Western blot analysis of oxidant-damaged proteins in brain homogenates of 120 days old APPPS1;LMP7^+/+^ and respective littermate APPPS1;LMP7^−/−^ mice, as well as age-matched wild-type and LMP7^−/−^ mice using carbonyl-detection and (**k**) corresponding densitometric quantification (*n* = 2–4 mice per group; LMP7^+/+^ vs. LMP7^−/−^: *p* = 0.0096; LMP7^+/+^ vs. APPPS1;LMP7^+/+^: *p* = 0.0066; LMP7^+/+^ vs. APPPS1;LMP7^−/−^: *p* = 0.0023; ** *p* < 0.01, one-way ANOVA followed by Bonferroni post-tests). (nd = not detected and na = not analyzed)

Since previous data suggested an increase in iP gene expression during aging and in plaque-associated glia cells in APP/PS1 mice [41], we analyzed protein expression of LMP7 upon aging and in association with development of Aβ-pathology in brains of APPPS1 mice that show a more rapid Aβ-pathology than the APP/PS1 mice used in previous studies. While protein levels of LMP7 in brain homogenates were elevated during aging (from 49 to 250 days old mice, respectively) in wildtype littermate mice (LMP7^+/+^: 49d vs. 250d; *p* = 0.0253; two-way ANOVA followed by Bonferroni post-tests), this induction was significantly enhanced by the presence of Aβ-pathology in APPPS1 mice (LMP7^+/+^ vs. APPPS1;LMP7^+/+^: 49d ns; 120d *p* = 0.0222; 250d *p* = 0.0065; two-way ANOVA followed by Bonferroni post-tests) (Fig. 1a–c). These changes in protein expression were reflected in alterations in total chymotryptic-like activity of the proteasome (standard proteasomes and iPs) (Fig. 1d) that was found to be significantly affected by Aβ-pathology in 250 days old APPPS1 mice (LMP7^+/+^ vs. LMP7^−/−^: *p* = 0,0061 and APPPS1;LMP7^+/+^ vs. APPPS1;LMP7^−/−^: *p* = 0.0001; two-way ANOVA followed by Bonferroni post-tests) in which plaque pathology was fully established. For the latter age group, LMP7 deficiency resulted in significantly reduced chymotryptic-like activity, which can be accounted by the lack of functional iPs in the LMP7 deficient mice. Aβ-related changes in iP subunit protein levels and function were paralleled by increased gene expression of the *PSMB8* (encoding for LMP7) (120d: LMP7^+/+^ vs. APPPS1;LMP7^+/+^: *p* = 0.0020, and 250d: LMP7^+/+^ vs. APPPS1;LMP7^+/+^: *p* = 0.0162; two-way ANOVA followed by Bonferroni post-tests) and also another iP subunit gene *PSMB9* (encoding for LMP2) at 120 and 250 days of age, respectively (Fig. 1e) (120d: LMP7^+/+^ vs. APPPS1;LMP7^+/+^: *p* = 0.004 and 250d: LMP7^+/+^ vs. APPPS1;LMP7^+/+^: *p* = 0.0014; two-way ANOVA followed by Bonferroni post-tests). In contrast, there was no change in the expression of *PSMB10* (encoding for β2i/ Mecl-1).

To get a an insight in the regulation of iP subunit genes, we examined the transcription of other interferon-stimulated genes, namely of the IFN-stimulated gene 15 (*isg15*) and of the IFN-inducible protein 10 gene (*cxcl10*), both acting as chemokines in the brain and serve as indicators for type I IFN signaling. We found that their expression was not significantly affected by aging (Fig. 1f), while Aβ-pathology enhanced transcription of these genes significantly at 120 days of age (*isg15*: LMP7^+/+^ vs. APPPS1;LMP7^+/+^: *p* = 0.0165, and *cxcl10*: LMP7^+/+^ vs. APPPS1;LMP7^+/+^: *p* = 0.0007; two-way ANOVA followed by Bonferroni post-tests) indicating that increased iP expression and function are associated with exacerbated IFN-signaling in the brains of APPPS1 mice (Fig. 1f). Indeed, analysis of gene expression levels of type I interferons, namely *ifn-α* and *ifn-β* revealed a transient upregulation of their transcription at 120 days of age (*ifn-β*: LMP7^+/+^ vs. APPPS1;LMP7^+/+^: *p* = 0.0410; two-way ANOVA followed by Bonferroni post-tests) (Fig. 1g). Thus, increased iP expression in APPPS1 mice is most likely driven by type I IFN signaling.

IFN-signaling as well as AD have been shown to induce oxidative damage to proteins. Since oxidatively damaged proteins tagged by poly-ubiquitin conjugates are a target for iPs [49], we analyzed the levels of poly-ubiquitinylated proteins by Western blot. As expected, we observed a significant increase of poly-ubiquitinylated proteins upon development of Aβ-pathology in APPPS1;LMP7^+/+^ mice compared to wild-type littermate control animals. However, the deficiency in iP subunits did not affect the levels of poly-ubiquitinylated proteins in APPPS1;LMP7^−/−^ mice (Fig. 1h and i) suggesting an upregulation of standard proteasome (sP) activity during the development of Aβ-pathology. Since iPs are reported to be essential for cell viability, the amount of oxidant damaged proteins were analyzed indirectly by Western blot using an anti-DNP antibody directed against ROS- changed carbonyl groups of proteins. Indeed, iPs are essential for cell viability as the amount of irreversibly damaged proteins is significantly increased upon iP subunit deficiency in LMP7^−/−^ mice compared to LMP7^+/+^ littermate control mice (Fig. 1j and k). However, the concentration of damaged proteins is not further changed during Aβ-pathology.

### LMP7 deficiency does not affect Aβ-pathology in APPPS1 mice

**Fig. 2:**
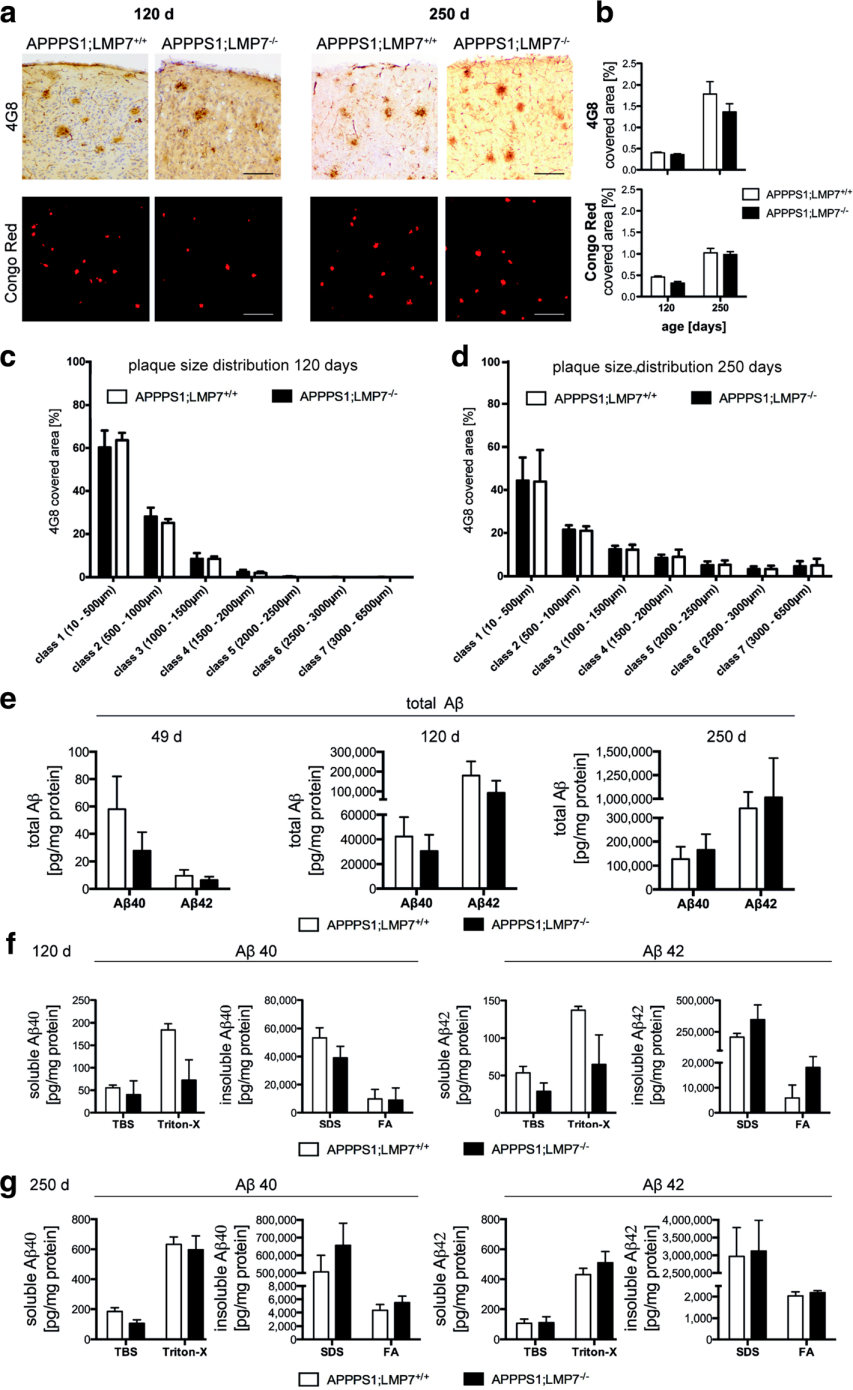
iP subunit deficiency has no impact on cerebral Aβ burden in young and aged APPPS1 mice. **a** Representative images of diffuse plaques (4G8 antibody immunohistochemistry) and congophilic core plaques assessed by Congo Red staining in cortical sections of young and aged APPPS1;LMP7^+/+^ and respective littermate APPPS1;LMP7^−/−^ mice (scale bar 100 μm) and (**b**) corresponding stereomorphological quantification of Aβ plaque burden (*n* = 4–8 mice per group, *p* > 0.05, Mann-Whitney test). **c** Plaque size distribution analysis using the CellSense software in the cerebral cortex of 120 days old APPPS1;LMP7^+/+^ and respective littermate APPPS1;LMP7^−/−^ mice and (**d**) aged 250 days old APPPS1;LMP7^+/+^ and respective littermate APPPS1;LMP7^−/−^ mice (*n* = 3–5 mice per group, *p* > 0.05, two-way ANOVA, followed by Bonferroni post-tests). **e** Total amounts of soluble and insoluble Aβ_1–40_ and Aβ_1–42_ species in brain homogenates of pre-plaque depositing, young 120 days old and aged250 days old APPPS1;LMP7^+/+^ and respective littermate APPPS1;LMP7^−/−^ mice assessed by MSD 96-Well MULTI-SPOT® Human (6E10) Abeta Triplex Assay (Meso Scale Discovery) (*n* = 4–8 mice per group, *p* > 0.05, one-way ANOVA followed by Bonferroni post-tests). **f** and (**g**) Amount of soluble and insoluble Aβ_1–40_ and Aβ_1–42_ species calculated from individual protein fractions assessed by MSD 96-Well MULTI-SPOT® Human (6E10) Abeta Triplex Assay (Meso Scale Discovery) in brain homogenates of (**f**) young 120 days old APPPS1;LMP7^+/+^ and respective littermate APPPS1;LMP7^−/−^ mice (*n* = 4–6 mice per group, *p* > 0.05, Mann-Whitney test) and (**g**) aged 250 days old APPPS1;LMP7^+/+^ and respective littermate APPPS1;LMP7^−/−^ mice (*n* = 5 mice per group, *p* > 0.05, Mann-Whitney test)

**Fig. 3:**
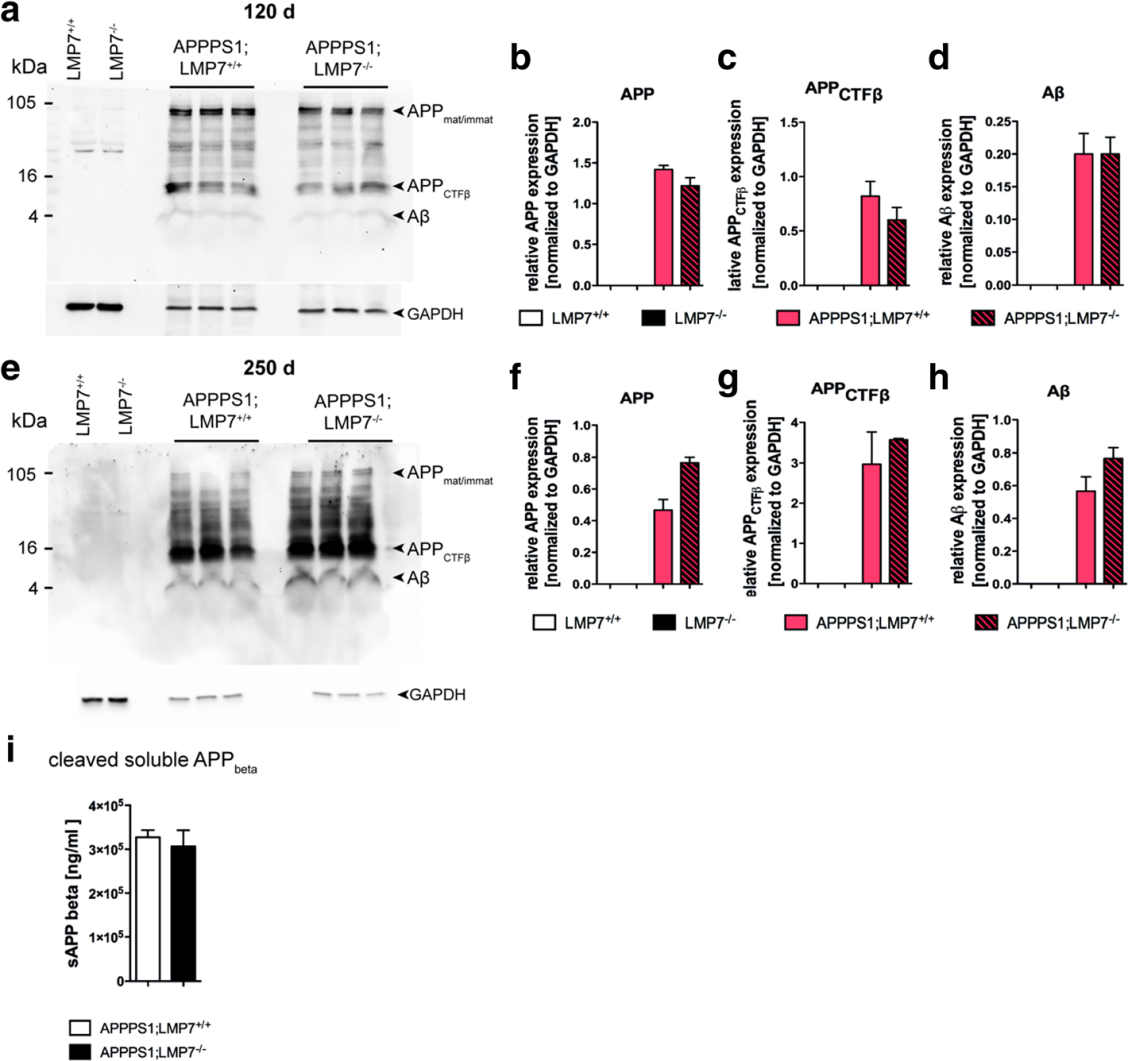
iP subunit deficiency has no impact on APP processing, on cerebral Aβ production and sAPPβ levels in brain homogenates in APPPS1 mice. **a** and (**e**) Representative image of Western blot analysis of APP, APP_CTFβ_ and Aβ in brain homogenates of young 120 days old (**a**; upper panel) and aged 250 days old (**e**; lower panel) APPPS1;LMP7^+/+^ and respective littermate APPPS1;LMP7^−/−^ mice (6E10 detection) and corresponding densitometric quantification (upper panel; **b** ***–*** **d** ***,*** and lower panel; **f** ***–*** **h**) (*n* = 4–6 mice per group, *p* > 0.05, Mann-Whitney test). **i**, Soluble APP_beta_ levels in Tris fractions of 49 day old pre-depositing APPPS1;LMP7^+/+^ and respective littermate APPPS1;LMP7^−/−^ mice assessed by Human Swedish Soluble APPβ (sw sAPPβ) singleplex Meso Scale assay (Meso Scale Discovery) (*n* = 4 mice per group, *p* > 0.05, Mann-Whitney test)

To test the potential impact of the observed increase in iP expression levels on Aβ-plaque pathology in vivo, we assessed the development and progression of Aβ-pathology in presence or absence of iP activity in APPPS1 mice harboring or lacking LMP7. Stereological quantification revealed no difference in Aβ plaque burden in brains of APPPS1 mice with or without functional LMP7 during the onset of Aβ-pathology (120 days of age; Fig. 2a and b; left panel), as well as in aged APPPS1 mice exhibiting extensive Aβ-pathology (250 days of age, Fig. 2a and b, right panel). In addition, the distribution of plaque sizes was similar in both experimental groups (Fig. 2c and d). Biochemical analyses of total Aβ-levels (Fig. 2e) as well as detailed analyses of soluble and insoluble Aβ-species (Fig. 2f and g) corroborated these findings. Moreover, expression of the amyloid precursor protein (APP) and APP processing were unaltered in APPPS1 mice lacking or harboring LMP7 (Fig. 3a–h). Furthermore, the amyloidogenic APP β-C terminal fragment released from APP by BACE cleavage remained unchanged (Fig. 3i). These data demonstrate that iP deficiency does not alter the development of Aβ-pathology in APPPS1 mice, does not affect size and solubility of Aβ-deposits and does not affect APP processing.

### Microglia activity and pro-inflammatory cytokine levels are reduced by iP deficiency in APPPS1 mice

**Fig. 4:**
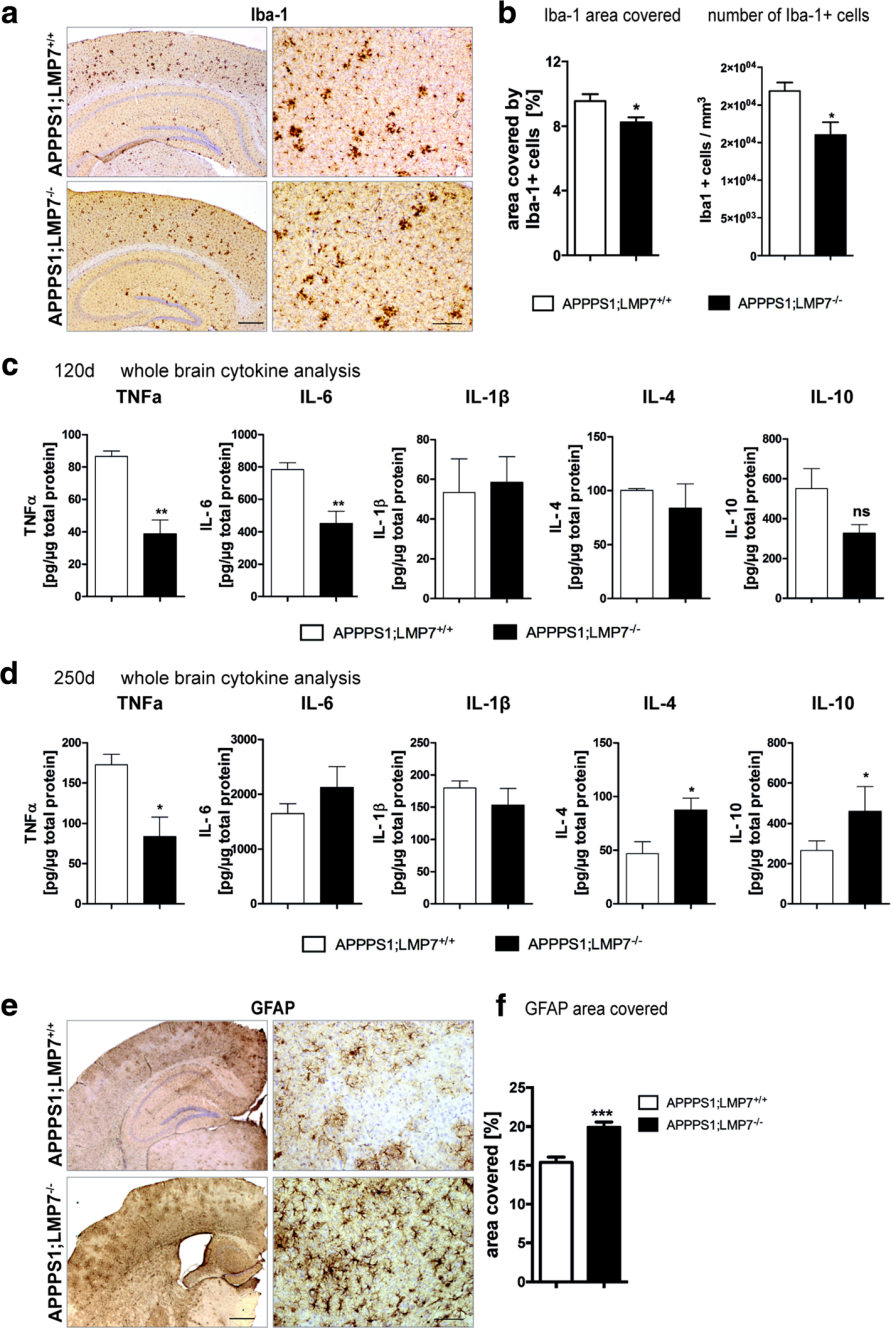
iP subunit deficiency changes microgliosis and the inflammatory milieu in APPPS1 mice. **a** Representative images of Iba-1 immunohistochemistry in cortical sections of 120 days old APPPS1;LMP7^+/+^ and respective littermate APPPS1;LMP7^−/−^ mice (left panels: scale bar 500 μm; right panels: scale bar 100 μm). **b** Corresponding stereomorphological quantification of the Iba-1 area covered and numbers of Iba-1+ cells at 120 days of age (*n* = 4–5 mice per group, area covered: APPPS1.LMP7^+/+^ vs. APPPS1;LMP7^−/−^: *p* = 0.0135; number of Iba + microglia cells: APPPS1.LMP7^+/+^ vs. APPPS1;LMP7^−/−^: *p* = 0.0286; unpaired t-test, two-tailed). **c** Analysis of whole brain cytokine levels assessed by Meso Scale V-PLEX Plus Pro-inflammatory Panel 1 (mouse) kit in brain homogenates of 120 days of age APPPS1;LMP7^+/+^ and respective littermate APPPS1;LMP7^−/−^ mice (*n* = 4–5 mice per group, TNFα: APPPS1.LMP7^+/+^ vs. APPPS1;LMP7^−/−^: *p* = 0.0079; IL-6: APPPS1.LMP7^+/+^ vs. APPPS1;LMP7^−/−^: *p* = 0.0079, Mann-Whitney test). **d** Analysis of whole brain cytokine levels assessed by Meso Scale V-PLEX Plus Pro-inflammatory Panel 1 (mouse) kit in soluble brain homogenates of 250 days of age APPPS1;LMP7^+/+^ and respective littermate APPPS1;LMP7^−/−^ mice (*n* = 4–5 mice per group, TNFα: APPPS1.LMP7^+/+^ vs. APPPS1;LMP7^−/−^: *p* = 0.0260; IL-4: APPPS1.LMP7^+/+^ vs. APPPS1;LMP7^−/−^: *p* = 0.0223; IL-10: APPPS1.LMP7^+/+^ vs. APPPS1;LMP7^−/−^: *p* = 0.0317, Mann-Whitney test). **e** Representative images of GFAP–positive astrocytes detected by immunohistochemistry in cerebal cortical sections. **f** Stereomorphological quantification of the GFAP area covered in APPPS1.LMP7^+/+^ and respective littermate APPPS1;LMP7^−/−^ mice (*n* = 5 mice per group, *** *p* = 0.001, two-tailed Student’s t-test)

Next we analyzed the effect of LMP7 deficiency on inflammation in APPPS1 mice. Since iP activation upon Aβ-pathology has been described mainly in glial cells, we analyzed the effect of iP deficiency on microgliosis in APPPS1 mice by stereologically analyzing microglia cells (Fig. 4a and b). Despite there being no difference in Aβ plaque burden, iP deficient APPPS1 mice displayed a significant reduction in the number of and percentage of area covered by Iba-1+ microglia cells compared to APPPS1 mice with functional iPs (Fig. 4a and b *,* area covered: APPPS1.LMP7^+/+^ vs. APPPS1;LMP7^−/−^: *p* = 0.0135; unpaired t-test, two-tailed and number of Iba + microglia cells: APPPS1.LMP7^+/+^ vs. APPPS1;LMP7^−/−^: *p* = 0.0286; unpaired t-test, two-tailed), indicating that iP deficiency changes the microglial response to Aβ-pathology. The slight reduction in the number of microglia in APPPS1 mice lacking LMP7 compared to those harboring LMP7 was accompanied by changes in the concentration of pro-inflammatory secreted cytokines in vivo (Fig. 4c and d), with a significant reduction of TNFα and IL-6 levels (TNFα: APPPS1.LMP7^+/+^ vs. APPPS1;LMP7^−/−^: *p* = 0.0079 and IL-6: APPPS1.LMP7^+/+^ vs. APPPS1;LMP7^−/−^: *p* = 0.0079, Mann-Whitney test, two-tailed), while IL-1β, IL-4 and IL-10 levels remained unchanged at 120 days of age (Fig. 4c). However, this cytokine profile changed upon aging and prolonged Aβ exposure. Aged (250 days old) APPPS1 mice deficient in iPs still displayed a significant reduction of TNFα in soluble brain extracts (TNFα: APPPS1.LMP7^+/+^ vs. APPPS1;LMP7^−/−^: *p* = 0.0260; Mann-Whitney test, two-tailed), while IL-6 levels were unchanged. In contrast, the anti-inflammatory cytokines IL-4 and IL-10 levels were significantly increased in soluble brain extracts of aged LMP7-deficient APPPS1 mice (250 days old; Fig. 4d *,* (IL-4: APPPS1.LMP7^+/+^ vs. APPPS1;LMP7^−/−^: *p* = 0.0223 and IL-10: APPPS1.LMP7^+/+^ vs. APPPS1;LMP7^−/−^: *p* = 0.0317; Mann-Whitney test, two-tailed). The observed change in the microglia compartment was accompanied by an increase in astrogliosis in iP deficient APPPS1 mice compared to iP competent APPPS1 mice (Fig. 4e and f, area covered by GFAP+ astrocytes, APPPS1.LMP7^+/+^ vs. APPPS1;LMP7^−/−^: *n* = 5 mice per group, *** *p* = 0.001, two-tailed Student’s t-test), most likely secondary to the changes in microglia response and cytokine secretion. Taken together, our results indicate a role for iPs in modulating the glial response during the course of Aβ-pathology in APPPS1 mice.

### Microglia deficient in LMP7 subunits elicit attenuated cytokine responses in vitro

**Fig. 5:**
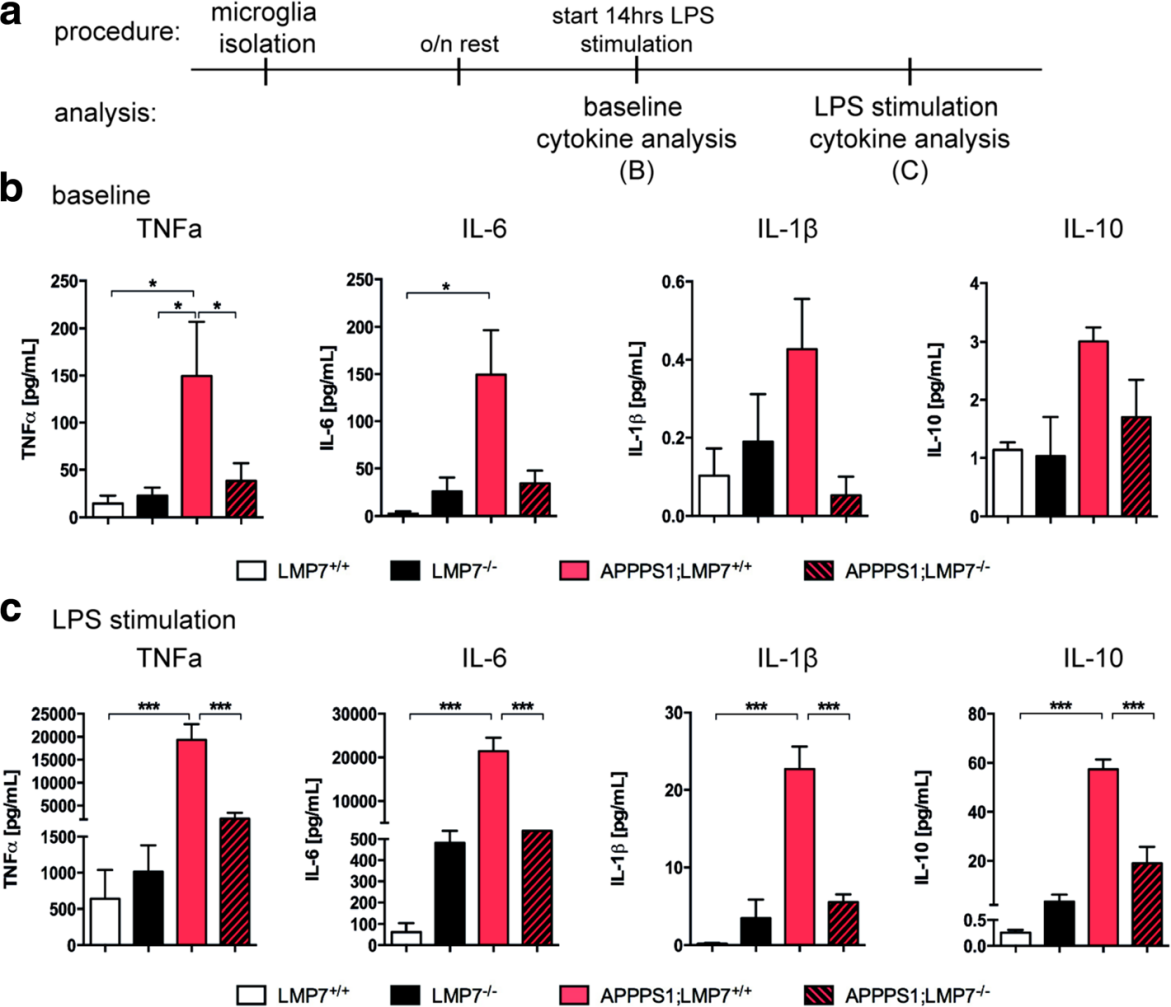
iP subunit deficiency alters cytokine secretion profile in adult microglia in vitro. **a** Schematic representation of experimental procedure for microglia isolation, LPS stimulation and cytokine analyses. **b** Cytokine levels in supernatant of cultured enriched microglia form 120d old mice at baseline assessed by Meso Scale V-PLEX Plus Pro-inflammatory Panel 1 (mouse) kit (Meso Scale Discovery) (*n* = 3–4 wells per group, TNFα: LMP7^+/+^ vs. APPPS1;LMP7^+/+^: *p* = 0.0275; LMP7^−/−^ vs. APPPS1;LMP7^+/+^: *p* = 0.0394; APPPS1;LMP7^−/−^ vs. APPPS1;LMP7^+/+^: *p* = 0.0252; IL-6: LMP7^+/+^ vs. APPPS1;LMP7^+/+^: *p* = 0.0095; LMP7^−/−^ vs. APPPS1;LMP7^+/+^: *p* = 0.0307; APPPS1;LMP7^−/−^ vs. APPPS1;LMP7^+/+^: *p* = 0.0471; one-way ANOVA followed by Bonferroni post-tests). **c** Cytokine levels in supernatant of cultured enriched microglia isolated from 120d old mice after 14 h LPS stimulation assessed by Meso Scale V-PLEX Plus Pro-inflammatory Panel 1 (mouse) kit (Meso Scale Discovery) (*n* = 3–4 wells per group, TNFα: LMP7^+/+^ vs. APPPS1;LMP7^+/+^: *p* = 0.0002; LMP7^−/−^ vs. APPPS1;LMP7^+/+^: *p* = 0.0002; APPPS1;LMP7^−/−^ vs. APPPS1;LMP7^+/+^: *p* = 0.003; IL-6: LMP7^+/+^ vs. APPPS1;LMP7^+/+^: *p* = 0.0002; LMP7^−/−^ vs. APPPS1;LMP7^+/+^: *p* < 0.0001; APPPS1;LMP7^−/−^ vs. APPPS1;LMP7^+/+^: *p* < 0.0001; IL-1β: LMP7^+/+^ vs. APPPS1;LMP7^+/+^: *p* < 0.0001; LMP7^−/−^ vs. APPPS1;LMP7^+/+^: *p* < 0.0001; APPPS1;LMP7^−/−^ vs. APPPS1;LMP7^+/+^: *p* = 0.0001; IL-10: LMP7^+/+^ vs. APPPS1;LMP7^+/+^: *p* < 0.0001; LMP7^−/−^ vs. APPPS1;LMP7^+/+^: *p* < 0.0001; APPPS1;LMP7^−/−^ vs. APPPS1;LMP7^+/+^: *p* = 0.0002; one-way ANOVA followed by Bonferroni post-tests)

Since we observed a modulation of microglia activity in APPPS1 mice deficient in LMP7 in vivo, we examined the effect of iP deficiency on microglia cytokine release in vitro by culturing microglia isolated from mouse brain tissues (Fig. 5a). As expected, primary microglia isolated from 120 day old APPPS1 mice exhibited significantly elevated levels of the pro-inflammatory cytokines TNFα and IL-6 at baseline compared to wildtype littermate control animals (Fig. 5b *,* TNFα: LMP7^+/+^ vs. APPPS1;LMP7^+/+^: *p* = 0.0275; LMP7^−/−^ vs. APPPS1;LMP7^+/+^: *p* = 0.0394; APPPS1;LMP7^−/−^ vs. APPPS1;LMP7^+/+^: *p* = 0.0252; IL-6: LMP7^+/+^ vs. APPPS1;LMP7^+/+^: *p* = 0.0095; LMP7^−/−^ vs. APPPS1;LMP7^+/+^: *p* = 0.0307; APPPS1;LMP7^−/−^ vs. APPPS1;LMP7^+/+^: *p* = 0.0471; one-way ANOVA followed by Bonferroni post-tests). While iP deficiency tended to increase microglia cytokine release in the non-pathological context, microglia isolated from APPPS1 mice deficient in iPs secreted significantly reduced amounts of pro-inflammatory cytokines TNFα and IL-6 (TNFα: APPPS1;LMP7^−/−^ vs. APPPS1;LMP7^+/+^: *p* = 0.0252 and IL-6: APPPS1;LMP7^−/−^ vs. APPPS1;LMP7^+/+^: *p* = 0.0471; one-way ANOVA followed by Bonferroni post-tests), whereas levels of IL-1β and IL-10 were only slightly affected (Fig. 5b). The cytokine profiles observed in isolated microglia in vitro corresponded to the cytokine levels detected in whole brain homogenates in vivo (Fig. 4c and d). After additional stimulation with LPS to fully induce iP expression, secretion of all measured cytokines was significantly attenuated in APPPS1 mice lacking LMP7 compared to microglia isolated from iP competent APPPS1 mice (Fig. 5c *,* TNFα: LMP7^+/+^ vs. APPPS1;LMP7^+/+^: *p* = 0.0002; LMP7^−/−^ vs. APPPS1;LMP7^+/+^: *p* = 0.0002; APPPS1;LMP7^−/−^ vs. APPPS1;LMP7^+/+^: *p* = 0.003; IL-6: LMP7^+/+^ vs. APPPS1;LMP7^+/+^: *p* = 0.0002; LMP7^−/−^ vs. APPPS1;LMP7^+/+^: *p* < 0.0001; APPPS1;LMP7^−/−^ vs. APPPS1;LMP7^+/+^: *p* < 0.0001; IL-1β: LMP7^+/+^ vs. APPPS1;LMP7^+/+^: *p* < 0.0001; LMP7^−/−^ vs. APPPS1;LMP7^+/+^: *p* < 0.0001; APPPS1;LMP7^−/−^ vs. APPPS1;LMP7^+/+^: *p* = 0.0001; IL-10: LMP7^+/+^ vs. APPPS1;LMP7^+/+^: *p* < 0.0001; LMP7^−/−^ vs. APPPS1;LMP7^+/+^: *p* < 0.0001; APPPS1;LMP7^−/−^ vs. APPPS1;LMP7^+/+^: *p* = 0.0002; one-way ANOVA followed by Bonferroni post-tests). These data demonstrate that the iP is involved in modulating microglia cytokine secretion in the context of Aβ-pathology and upon exogenous stimulation.

### Cognitive deficts are attenuated by iP deficiency in APPPS1 mice

To determine whether iP deficiency and its subsequent reduction in pro-inflammatory cytokines alters cognitive function in AD model mice, we performed a variety of behavioral tests with APPPS1 mice lacking or harboring LMP7.

**Fig. 6:**
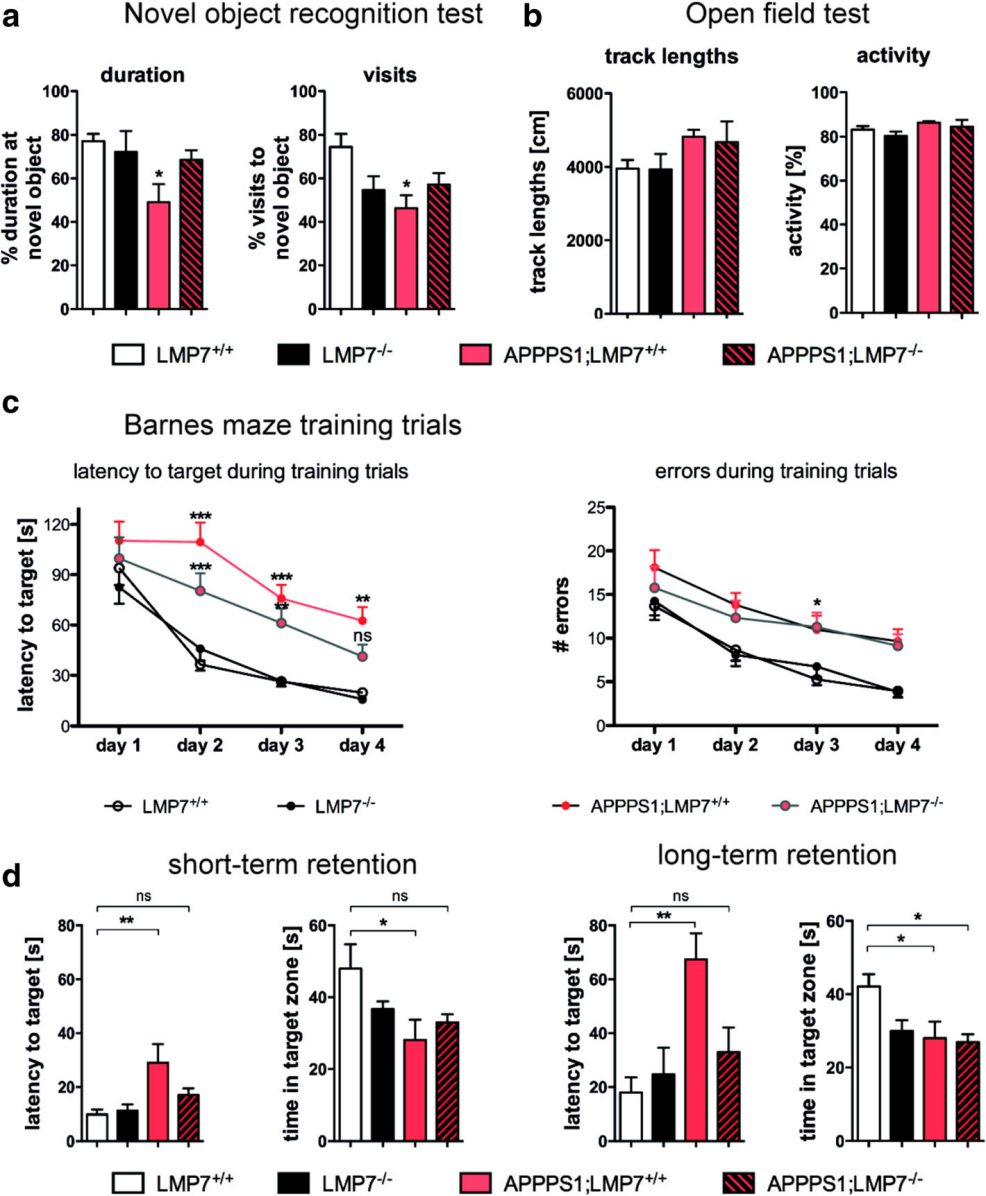
Improved cognitive function of APPPS1 mice upon iP deficiency. **a** Aged APPPS1;LMP7^+/+^ and respective littermate APPPS1;LMP7^−/−^ mice underwent behavioral tests for cognition, LMP7^+/+^ and LMP7^−/−^ mice served as controls. Percentage of time spent exploring the novel object and percentage of visits to the new object in the Novel object recognition test (*n* = 8–10 mice per group; duration: LMP7^+/+^ vs. APPPS1;LMP7^+/+^: *p* = 0.0475; visits: LMP7^+/+^ vs. APPPS1;LMP7^+/+^: *p* = 0.0209; one-way ANOVA followed by Bonferroni post-tests). **b** Quantification of recorded track lengths and respective mouse activity of APPPS1;LMP7^+/+^ and respective littermate APPPS1;LMP7^−/−^ mice analyzed in the Open field arena (*n* = 8–10 mice per group; p > p 0.05; one-way ANOVA followed by Bonferroni post-tests). **c** Latency to target and number of errors during training trials during the Barnes maze paradigm (*n* = 8–10 mice per group; day 4*:* LMP7^+/+^ vs. APPPS1;LMP7^+/+^: *p* = 0.0033; LMP7^+/+^ vs. APPPS1;LMP7^−/−^: *p* = 0.2096; two-way ANOVA followed by Bonferroni post-tests). **d** Short-term and long-term memory retention trail: Quantification of latency to reach the target and total time spent in the target zone during the Barnes maze paradigm (*n* = 8–10 mice per group; short-term retention: LMP7^+/+^ vs. APPPS1;LMP7^+/+^: *p* = 0.0067; LMP7^+/+^ vs. APPPS1;LMP7^+/+^: *p* = 0.0322; long-term retention: LMP7^+/+^ vs. APPPS1;LMP7^+/+^: *p* = 0.0022; APPPS1;LMP7^−/−^ vs. APPPS1;LMP7^+/+^: *p* = 0.0548; LMP7^+/+^ vs. APPPS1;LMP7^+/+^: *p* = 0.0322; LMP7^+/+^ vs. APPPS1;LMP7^−/−^: *p* = 0.0178; one-way ANOVA followed by Bonferroni post-tests)

When examining performance in the novel object test, a measure for cortical dependent memory function, APPPS1 mice deficient in iP activity showed increased exploration of and visits to the novel objects (Fig. 6a) compared to APPPS1 mice with functional iP activity (duration: LMP7^+/+^ vs. APPPS1;LMP7^+/+^: *p* = 0.0475; visits: LMP7^+/+^ vs. APPPS1;LMP7^+/+^: *p* = 0.0209; one-way ANOVA followed by Bonferroni post-tests), indicating improvement of cortical memory in iP deficient APPPS1 mice. Analysis of spatial memory performance in the Barnes maze test revealed normal learning and memory abilities of wildtype and iP deficient mice. APPPS1 mice with normal iP activity showed significant impairment in learning the task (Fig. 6c *,* day 4*:* LMP7^+/+^ vs. APPPS1;LMP7^+/+^: *p* = 0.0033; LMP7^+/+^ vs. APPPS1;LMP7^−/−^: *p* = 0.2096; two-way ANOVA followed by Bonferroni post-tests), while APPPS1 mice deficient in iPs showed a slight amelioration of these learning deficits. This was demonstrated by a reduced latency to locate the escape chamber during the short-term memory retention trial compared to cognitively-impaired APPPS1 mice with functional iP activity (Fig. 6d *,* short-term retention: LMP7^+/+^ vs. APPPS1;LMP7^+/+^: *p* = 0.0067 and LMP7^+/+^ vs. APPPS1;LMP7^−/−^: *p* = 0.0322; long-term retention: LMP7^+/+^ vs. APPPS1;LMP7^+/+^: *p* = 0.0022; APPPS1;LMP7^−/−^ vs. APPPS1;LMP7^+/+^: *p* = 0.0548; LMP7^+/+^ vs. APPPS1;LMP7^+/+^: *p* = 0.0322; LMP7^+/+^ vs. APPPS1;LMP7^−/−^: *p* = 0.0178; one-way ANOVA followed by Bonferroni post-tests), indicating an mild improvement of hippocampus-dependent memory impairment by iP deficiency in APPPS1 mice.

Taken together, these data indicate a beneficial effect of iP deficiency on cognitive performance in mice developing AD-like Aβ-pathology, which coincides with reduced secretion of pro-inflammatory cytokines upon modulation of iP function (Fig. 4a-e).

## Discussion

We herein demonstrate that elements of the proteasome system, namely iP subunits such as LMP7, are increased in the CNS upon aging and that this phenomenon is further accelerated by concomitant development of AD associated Aβ-pathology in APPPS1 mice, most likely mediated through type I interferon induction. Deletion of functional iPs in APPPS1 mice modulates the Aβ-associated inflammatory signature resulting in a mild ameloriation of the pathology-associated behavioral phenotype, which is in line with the data of the Green lab [13, 51].

The proteasome system consisting of various isoforms is a major defense mechanism against pathologic changes in proteostasis and essential for cellular integrity. Thus, it is expressed in all cells, including neurons, microglia and astrocytes in the brain [31]. However, the expression and activity of the various proteasome isoforms varies between cell types. Induction of iP expression was detected in cultured and IFNγ treated microglia in vitro [52] as well as in plaque associated microglia and astrocytes in vivo [41]. Whereas standard proteasome (sP) activity is reduced in aging [26] and in neurodegenerative disorders [27, 48] possibly leading to protein aggregation, oxidation and neuronal degeneration, the iP has been observed to be upregulated in human brains in the context of ageing, AD and Huntington’s disease [16, 37]. However, the impact of in vivo iP inhibition or deficiency on the development and progression of neurodegenerative diseases such as AD has not been studied to date.

Rodent studies have generated inconsistent data showing decreased, unaltered and increased proteasomal capacity during the course of aging [20, 57]. In the context of AD, iP activity was shown to be impaired in a mouse model exhibiting Aβ-pathology [2], despite increased expression of iP subunits. In line with the latter finding, we also demonstrate herein that expression levels of β5i/LMP7/*PSMB8* iP subunits are increased upon aging, which are further enhanced by concomitant deposition of Aβ in mice exhibiting AD-like pathology. This is also true for the expression of the β1i/LMP2/*PSMB9* gene encoding another IP subunit. Besides the changes in gene expression, we can also show that the chymotryptic-like activity is increased due to increased Aβ-pathology, which is in accordance with a study by Orre and colleagues demonstrating that proteasomal activity is upregulated in APP/PS1 mice [41]. It is very likely that the choice of animal models, of assays used to measure iP activity, and the choice of time point of analysis account for some of the discrepancies regarding changes in proteasome activity in previously published studies. In our present study the induction of iP subunits coincided with the transient upregulation of type I IFN and other IFN-stimulated genes, which represents a likely signaling pathway for the induction of iP subunit expression. The induction of type I IFN-production and the chemokine CXCL-10 was previously reported in cells treated with proteasome inhibitors or in PRAAS patient’s cells indicating that Aβ deposition impairs proteasome function [8]. Furthermore, the axis of CXCL-10 and its receptor CXCR3 expressed on microglia have been implicated in promoting plaque formation and behavioral deficits in APPPS1 mice [30]. In addition, ablation of type I IFN signaling has been shown to preserve cognitive function and cytokine pattern in a mouse model of AD [36].

The LMP7 deficient mouse model was initially used to study the impact of iPs in antigen presention via MHC I molecules. In addition, more recent studies demonstrating a pathogenetically relevant contribution of iPs in inflammation-driven diseases [7, 17, 40, 49] not only extended our knowledge on the role of iP, but provided yet another rational to investigate their role in the pathogenesis of AD, since inflammation-mediated processes in AD are known to partake in disease progression. Deficiency of iP function did not substantially impact Aβ plaque burden and soluble Aβ levels in APPPS1 mice, although there was a trend to reduced soluble Aβ levels at an early disease stage (120d). One possible explanation is that iP-deficient mice may adapt to their loss of iP activity by upregulation of sP activity. Moreover, APPPS1 mice are known to overexpress toxic Aβ species rapidly and at very high levels, thus eventually overriding rather small effects of iP deficiency on Aβ plaque pathology.

Microglia are known to be key in promoting the pathogenetically relevant contribution of the immune system in AD by a vast release of inflammatory molecules. Secretion of pro-inflammatory cytokines by microglia and associated changes in phagocytic and neuroprotective properties are a major contributing factor to the recently recognized “cellular” phase of Alzheimer’s disease [14]. Upon deleting iPs in APPPS1 mice we observed changes in cytokine secretion of microglia which are likely due to an altered control of regulatory factors of the nuclear factor-κ B (NFκB) family that are important for cytokine release [4, 6, 9, 39, 53]. In line with our observation in the CNS, selective inhibition of β5i/LMP7 in activated peripheral blood mononuclear cells (PBMCs) led to a downregulation of cytokine production via the NFκB pathway [39]. LPS-induced signaling pathways were also significantly reduced in peritoneal macrophages lacking immunoproteasome subunits [46] indicating an important role for the iP in modulating the NFκB signaling pathway and therefore cytokine release. Specifically, increased activity of iPs results in enhanced degradation of NFκB inhibitor α (IkBα), which in turn is more stable in the context of iP-deficiency [40, 49].

The observed changes in microglial activation and cytokine secretion upon β5i/LMP7/*PSMB8* deficiency were accompanied by an increase in activated astrocytes. Since microglial cytokines are known to modulate the activation state of astrocytes [32, 50], these changes are most likely secondary to the altered microglial cytokine secretion profile, although we cannot exclude a direct effect of β5i/LMP7/*PSMB8* deficiency on astrocytes.

While CNS-derived soluble immune factors including pro-inflammatory cytokines like IL-1β, TNFα and IL-6 have been studied intensely [10, 11, 21, 23, 29, 52, 55], their role in AD is still a matter of debate, also due to the fact that the respective results - at least for some of the analyzed cytokines - is not always consistent [43]. Several studies altered cytokine levels through genetic manipulation of pro- and anti-inflammatory molecules with the aim of modulating chronic inflammation and altering Aβ plaque burden in transgenic AD-like mice [10, 11, 21, 23, 29, 55]. The diverse role of cytokines in the progression of AD-like pathology is underscored by recent observations showing that overexpression of the anti-inflammatory cytokine IL-10 negatively affected cognitive function [11], whereas IL-10 deficiency significantly restored cognitive impairment of APPPS1 mice [23]. In line with these later studies, we observed that reduced pro-inflammatory cytokine levels upon iP deficiency in APPPS1 mice were associated with improved cognitive performance, although this recovery in cognition was not accompanied by detectable changes in Aβ plaque pathology. Similarly, pharmacological elimination of microglia resulting in amelioration of cognitive deficits and reducing pro-inflammatory cytokines in AD-like mice did not translate into changes in plaque burden [13, 51]. Since the amount of amyloid plaque burden in AD subjects does not necessarily correlate with the level of functional deficits, this further supports the notion that cognitive performance and the amount of amyloid burden do not need to correlate inevitably [5, 47]. On the other hand, studies in AD patients suggest that elevated levels of pro-inflammatory cytokines are associated with impaired cognitive function [10, 11, 21, 35, 55] and highlight the potential of manipulation of this pro-inflammatory response for improving cognitive function without necessarily targeting Aβ deposition.

## Conclusion

In conclusion, our data demonstrate that iP deficiency is associated with reduced pro-inflammatory cytokine secretion in vitro and in vivo independent of a major impact on Aβ-pathology in a mouse model of AD. The reduction of pro-inflammatory cytokine secretion was accompanied by improvement of Aβ-pathology associated cognitive deficits, indicating that manipulation of iP activity may be a novel strategy to manipulate cytokine signaling and improve cognitive function in AD independent of targeting Aβ deposition. Furthermore, the development of selective iP inhibitors [39] may allow fine-tuned manipulation of cytokine signaling pathways to make this a viable option for targeting the innate immune response in AD and other neurodegenerative diseases.
